# The Negative Effects of Long Time Physical Activity Calorie Equivalent Labeling on Purchase Intention for Unhealthy Food

**DOI:** 10.3390/ijerph19063463

**Published:** 2022-03-16

**Authors:** Yuanhao Huang, Xiaoke Yang, Qian Chen

**Affiliations:** 1 School of Business, Renmin University of China, Beijing 100089, China; 2019000720@ruc.edu.cn; 2 School of Internet Economics and Business, Fujian University of Technology, Fuzhou 350002, China; 3 College of Management and Economics, Fujian Agriculture and Forestry University, Fuzhou 350002, China

**Keywords:** PACE labelling, unhealthy food, future self-continuity, purchase intention

## Abstract

(1) Background: Obesity has become a global epidemic that arouse much attention from governments, companies and scholar. Physical activity calorie equivalent (PACE) labels are introduced as a more effective nudge invention on less-calorie ordering. However, the effects of PACE labels are controversial in previous literature, thus, the research objective is to explore the effects of different PACE labels and furtherly to explore the underlying psychological mechanism; (2) Methods: Across four scenario-based experiments, involving potato chips, chocolate and cookies, this study manipulated the three calorie-information labeling (standard calorie label, long time PACE and short time PACE labels). Meanwhile, the mediating mechanism of the effects involving anticipatory guilt and the moderation effects between consumers’ future self-continuity and PACE labels are also measured; (3) Results: Results show that compared with the short time PACE and calorie labels, the longtime PACE labels have more negative influence on consumers’ purchase intention for unhealthy food. What’s more, the anticipatory guilt has negative effect of PACE labels as consumers are often prone to feeling guilty in the process of unhealthy food consumption. In addition, individuals with high future self-continuity have higher self-control and take more consideration of future outcomes, they are reluctant to choose unhealthy food than others; (4) Conclusions: Unhealthy food with a long time PACE label has more negative effect on consumers’ purchase intention rather than a short time PACE label. At the same time, companies that produce healthy foods should actively participate in the movement to label calories through the PACE labels.

## 1. Introduction

Healthy eating is of great importance, as unhealthy eating habits have led to global obesity epidemic [1], which causes a number of chronic diseases, such as type 2 diabetes, dyslipidemia, cardiovascular disease and periodontal disease [2,3,4]. The consequences of obesity affect not only the individual health of citizens, but also government expenditure on health [5]. Previous literature has confirmed that one of the effective methods to prevent unsustainable eating habits is to reduce unhealthy foods [6], how to prevent excess energy intake has aroused many attentions from the governments and social organizations [7,8].

Nudge-interventions are new concepts based on behavioral economic theory [9], which is a strategy to change people’s behavior in a predictable way without prohibiting any options or significantly altering economic incentives [10]. One promising direction of nudge interventions is to promote healthier eating habits [11]. For example, food labeling can change people’s food-consumption behaviors through providing detail information about the food, and this strategy has been widely adopted by many countries [12,13]. In the United States, all the chain restaurants are required to labeled calorie information on menus so that people are encouraged to choose low-calorie foods [14]. However, the effects of calorie labeling on unhealthy food consumption are controversial [15,16]. Some studies have mentioned that consumers would fail to catch the point of caloric intake when much numerical information is provided [17]. On the other hand, current calorie labeling is not clear enough to help consumer reduce calorie intake [17].

**Figure 1:**
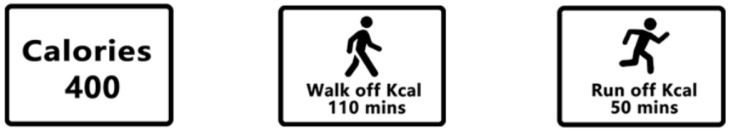
The examples of PACE labels (400 kcal).

Consumers spend around 6 s on scanning the front-of-pack labeling of foods [18], thus, an understandable form of labels is crucial to making change toward healthy diets [19]. According to Hersey et al. (2013), symbolic labels are more effective than numerical labels when perceived as nudge interventions [20]. A form of calorie labels called physical activity calorie equivalent (PACE) labels were proposed by the Royal Society for Public Health recently [21], which translates calories that the miles or minutes of different sports needed to burn off based on the consumption of a certain food item (as shown in Figure 1).

At present, studies on PACE labels can be summarized as the following directions: The first is the constraining effect of PACE labels on consumers’ calorie intake. Studies have shown that people ordered fewer calories from menus with exercise information, and 82% of participants preferred menus with physical activity labels [22]. In addition, parents who were shown the PACE labels no matter the minute- or mile-form, they ordered fewer calories for their children [23]. In addition, through multiple systematic reviews and meta-analyses, Daley et al. found that compared with other types of food labels or no food labels, the PACE label reduced the number of kilocalorie that consumers choose from the menu [24]. However, opposite results also shown in a study by Shah et al., who found that there were no differences in between PACE and calorie labels of food choices [25]. Seyedhamzeh et al., found that PACE labeling in minutes does not significantly reduce calories ordered compared to calorie-only labeling through meta-analyses [26]. In general, the effects of PACE labels are still open to debate.

In order to study the contradictory effects of the PACE labels deeply, scholars put emphasis on the different types of food which may cause inconsistent effects of PACE labels on consumer food consumption [16]. For example, PACE labels can reduce prospective food consumption of familiar snack foods, while the effects is opposite in the unfamiliar snack groups [27]. What’s more, another study suggests that the PACE labels can undermine the consumer preferences for unhealthy foods while consumer preferences for healthy foods are promoted by same labels [6]. Taken all, the effects of PACE labels should be addressed in specific food products, which can partly answer the question that PACE labels show controversial effects in former literature.

The third direction is concerned about the forms of PACE labels, which may show different effects on the purchase intention for unhealthy foods. For example, 370 kilocalories can be demonstrated by different time labels based on types of sports, such as walking for 35 min or running for 15 min. However, it is unclear which form (walking for a long time or running for a short time) is more likely to reduce consumers’ willingness to buy unhealthy foods. Few studies have explored such questions, Yang et al. (2021) found that minutes of walking to burn off the calories label has better effects on reducing consumers preferences for unhealthy foods [6]. However, the question still remains that this effect is robust or not, furthermore, the underlying logic behind this effect has not been explored. In this study, the effects of different PACE labels on consumers’ purchase intention for unhealthy food and the underlying psychological mechanism are explored. We try to work out the contradictory conclusions about the effects of PACE labels on the consumers’ purchase intention for unhealthy food.

In this study, “long time PACE labels” mean that it takes more time to burn off the calories with a moderate way (walking), while “short time PACE labels” represent less time is needed to burn off the calories with a vigorous way (running, swimming, rope skipping) [6]. In addition, experimental method is adopted to measure the effects of different PACE labeling forms and calorie label on the consumers’ purchase intention for unhealthy food accurately. Anticipatory guilt, which is used as a psychological variable to explain the psychological mechanism of discrepancy in effects of different PACE labels [28]. In addition, the moderating effect of consumers’ future self-continuity is also discussed in this study [29].

Based on the above discussion, the aims of this study are triple: (a) to estimate the effects of different forms of labeling (standard calorie label, long time PACE and short time PACE labels) on consumers’ purchase intention for unhealthy food; (b) to examine the mediating mechanism of the effects involving anticipatory guilt; (c) to determine the moderation effects between consumers’ future self-continuity and PACE labels.

## 2. Hypothesis Development

### 2.1. The Effects of PACE Labels on Unhealthy Food Products

The effects of PACE labels on unhealthy food consumption are still open to debate [30]. Few studies, though, have found that PACE labels have no effect on unhealthy food choices compared with calorie labels [16,25]. Many studies have concluded that PACE labels are more effective at reducing consumers’ choices of unhealthy food [22,23,31].

In a recent study of 570 respondents in a choice experiment method (CE), PACE labels are found to have different effects on consumers’ preferences for unhealthy food under different conditions [6]. To be specific, long time PACE labels have a stronger negative effect on consumers’ willingness to choose unhealthy food than calorie labels. In addition, short time PACE labels have a weak effect on consumers’ willingness to choose unhealthy food, and has no significant difference with calorie labels. However, CE method is to determine the correlation between different labels and the consumer preferences for unhealthy food. In this study, four experiments are used to verify causality between different PACE labels and the consumers’ purchase intention for unhealthy food while compared to other labels.

Why does long time PACE labels have a stronger negative effect when compared with short time PACE labels? We believe that long time PACE labels reflect a longer time to consume the calories, which can give consumers greater time pressure and calorie risk assessment [32]. On the contrary, short time PACE labels show more vigorous exercises, but less time is needed may cause consumers feel less potential calorie risk and time pressure, the effect is the same as calorie label. The stronger perception of potential calorie risk leads to a higher level of negative emotion predicted for unhealthy food [33], leading to a stronger negative effect on the consumers’ purchase intention for unhealthy food [34,35]. Therefore, we assumed that long time PACE labels can stimulate consumers’ risk perception of calories and time pressure, which result in consumers’ negative emotion towards unhealthy food and thereby reducing consumers’ purchase intention for unhealthy food. The following hypothesis is proposed:

**Hypothesis** **1** **(H1).** *Compared with short time PACE labels, long time PACE labels are more effective in reducing consumers’ purchase intention for unhealthy food*.

### 2.2. Anticipatory Guilt

Why does the higher time pressure and calorie risk of long time PACE labels lead to a lower willingness to buy unhealthy food? This study analyzes the psychological mechanism of this negative effects through anticipatory guilt. When consumers buying food, they are prone to be stimulated by positive or negative emotions [36]. Generally, consumers experience more positive emotions [37], such as consuming fried foods to acquire a good mood [38]. However, if consumers have certain health goals (e.g., losing weight) when consuming food, the risk factors of food (e.g., high calorie, high oil and high sugar, and so on.) will conflict with consumers’ health goals and cause their negative emotions. Most consumers feel guilty for eating a piece of cream cake when you need to lose weight because you can’t help it [39].

Guilt is a self-conscious negative emotion that consumers experience when they are aware of negative consequences associated with their actions [40,41]. As mentioned above, consumers are prone to feel guilty when they perceive the health risks are associated with food consumption. Furthermore, in contrast with other negative emotions, guilt is more of an emotional process that an individual associates responsibility with a specific action, thus requiring the perpetrator to take personal responsibility [42]. Thus, taking responsibility for an action is the main characteristic of guilt. Relevant studies have shown that consumers are often prone to feeling guilty in the process of food consumption [43], and feel self-blame for overeating or choosing unhealthy food [36]. At the same time, unhealthy food consumption is perceived as indulgent behavior, which suggests a lack of self-control, so that consumers are more likely to blame themselves and feel guilty [40,44].

Literature has confirmed that before actual food consumption, consumers will consider the consequences of food consumption based on own health goals in the imagined consumption stage [36,45]. Even consumers do not actually consume food, the sense of guilt is also involved in judging future consumption, which is named anticipatory guilt in this study [36]. When food information activates consumers’ anticipatory guilt, consumers are more likely to acquire a sense of self-control, thus reducing their consumption of the unhealthy food [46]. In other words, consumers may reduce anticipatory guilt by avoiding unhealthy food consumption [47]. Another study also shows that anticipatory negative emotions can affect consumers’ food consumption behavior [28]. In a food consumption scenario, external information can activate consumers’ emotions, including advertisement or symbols [48]. A former study has demonstrated that nutrition labels would affect consumers’ food purchase intention and got s sense of guilt [49].

In this study, we assume PACE labels are more prominent in presentation of calories and may activate consumers’ anticipatory guilt about unhealthy food consumption. Combining with the Hypothesis 1, PACE labels can activate consumers’ anticipatory guilt when selecting foods. At the same time, in order to reduce anticipatory guilt, consumers would reduce purchase intention for unhealthy food. Therefore, this paper proposes the following mediation mechanism hypothesis:

**Hypothesis** **2** **(H2).** *Compared with short time PACE labels, long time PACE labels will make consumers have higher anticipatory guilt about unhealthy food consumption, and then reduce consumers’ purchase intention for unhealthy foods. In other words, anticipatory guilt plays a mediating role in the influence of long time PACE labels on the purchase intention for unhealthy foods*.

### 2.3. Future Self-Continuity

People have different levels of anticipatory guilt on the same thing because of differences in characteristics [50,51]. For example, people on diet would feel more guilty than others when consuming foods [52]. Future self-continuity is one of the important characteristics of consumers, which is defined as an individual’s understanding of the continuity and consistency between his present self and his future self [29]. According to individual’s different time orientation perception, it can be divided into past self, present self and future self [53]. The present self and the future self are continuous, that is, future self-continuity [29]. Future self-continuity has an impact on various decision-making behaviors of consumers, such as moral behavior [54], personal achievement and health-related behavior [55,56]. What’s more, relevant studies have shown that future self-continuity can influence individuals’ exercise behavior [56], and individuals with high future self-continuity usually have higher self-control [55]. However, previous studies have not explored the influence of future self-continuity on behaviors detrimental to individuals’ long-term health, such as overeating, smoking and alcohol abuse, in particular, unhealthy food consumption a physically harmful behavior in the long run [57].

Related studies have proved that individuals with high future self-continuity have higher self-control and take more consideration of future outcomes, they are also sensitive to time and reduce the tendency to procrastinate [55,58]. Meanwhile, consumers with higher self-control ability, time sensitivity and future expectation may pay more attention to food consumption, which reflects in considering exercise demand caused by calories. As a result, they may have stronger anticipatory guilt [44]. On the contrary, individuals with lower future self-continuity have a weaker self-control [59], leading to lower self-responsibility judgments for indulgent behaviors and therefore lower anticipatory guilt over unhealthy food consumption. Taken all, we infer that different future self-continuity will affect consumers’ anticipatory guilt about unhealthy foods.

Therefore, in this study, we assume that different levels of future self-continuity will regulate the effects of long time PACE labels on anticipatory guilt and then affect consumers’ purchase intention for unhealthy food. Therefore, the following hypothesis is proposed:

**Hypothesis** **3** **(H3).** *The influence of long time PACE labels on reducing consumers’ purchase intention for unhealthy food is moderated by consumers’ future self-continuity. Consumers with high future self-continuity, long time PACE labels can exert a stronger impact on reducing consumers’ purchase intention of unhealthy food products compared with short time PACE labels. When consumers are in low future self-continuity, there is no significant difference between short time PACE and long time PACE labels in the impact on reducing consumers’ purchase intention for unhealthy foods.*

**Figure 2:**
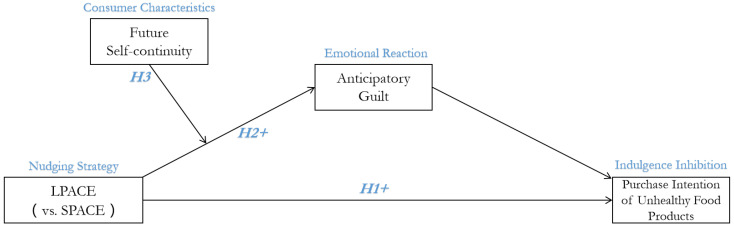
The main framework of the study.

The main framework of the present study was shown in Figure 2.

## 3. Materials and Methods

In order to verify three hypotheses, different stimulus materials are used in four scenario-based experiments (Table 1). Experimental method is the best way to prove the causal relationship between variables [60,61]. To eliminate interference caused by real-world environment [62], we mainly adopted situational simulation experiments. By changing the test items of the experiment, potential factors and alternative explanations are eliminated, improving the external and internal validity of research conclusions [63]. Finally, the data processing methods and processes we used are strictly in accordance with the standards of consumer experiments [64,65,66].

### 3.1. Study 1: Primary Examination of Main and Mediating Effects

The intergroup design of long time PACE labels (LPACE), short time PACE labels (SPACE), and calories label for comparison are used in this experiment. Our expectation was that LPACE has a stronger negative effect on the purchase intention for unhealthy food compared to the other two groups. However, there is no difference between the other two groups. In addition, we also verified that consumer preferences for different types of exercise did not affect the main effect of LPACE on purchase intention of unhealthy foods. Finally, in order to test whether the subjects really read the experimental stimulus and understood the content of the label, we asked the subjects to recall the label content of the stimulus and fill in the blanks at the end of the experiment, so as to exclude the careless subjects.

#### 3.1.1. Methodology

The Credamo online platform as a professional data survey platform is widely used to provide services for top universities around the world. In addition, it is also adopted as a tool of data collection for lots of authoritative journal articles [64,65,66,67]. In this research, 230 high-reliability respondents (users with a credit score above 90 on the Credamo) were randomly recruited from the Credamo and assigned to one of the following three experimental groups: LPACE, SPACE, and calories label. Through the random sample processing, the characteristics of items in different experimental groups were kept as consistent as possible to reduce the effect of demographic characteristics of items on the experimental results.

After checking whether there is a “Z” law, i.e., most of the items are the extreme value 1 or 7, or those fail to recall the labels. In this study, 14 samples were excluded and 216 valid questionnaires (the demographic characteristics of the specific samples were shown in Table 2) were collected, including 70 respondents in the LPACE group, 74 respondents in the SPACE group, and 72 respondents in the calories label group. According to the standards of consumer behavior experiment, the effective sample size of each group above 40 meets its requirements [6,68].

#### 3.1.2. Procedure and Materials

**Figure 3:**
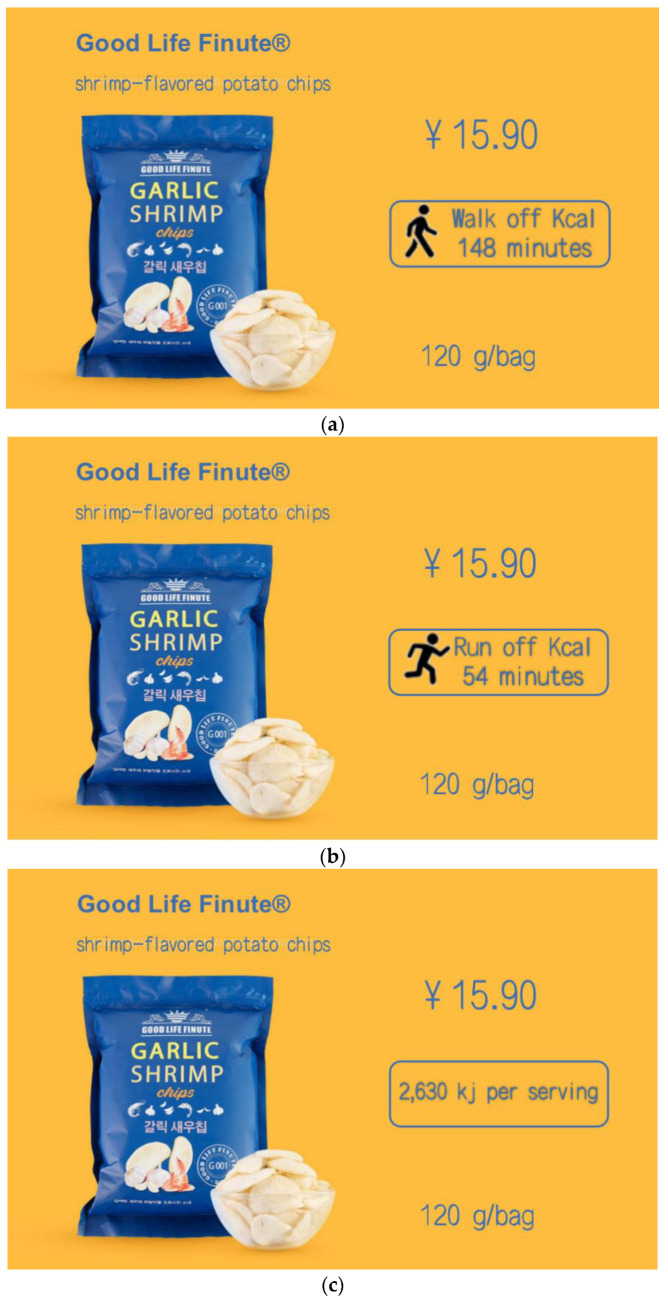
Stimuli of study 1. Note: (**a**) the LPACE group, (**b**) the SPACE group, and (**c**) the calories label group.

Participants were randomly assigned to one of these three groups. First of all, participants should read the basic information of shrimp-flavored potato chips, and accordingly evaluate and score it. The background information of the product in this experiment is that a food company is about to launch a new fictitious shrimp-flavored potato chips named Good Life Finute (120 g/bag, 15.9 yuan). The LPACE, SPACE and calories labels were attached to the product introduction of shrimp-flavored potato chips in these three groups, of which the general introduction was the same except for the difference in these three labels. The specific details of these three labels are as follows (Figure 3): LPACE means “it needs 148 min of walking to burn off the calories”; SPACE refers to “it needs 54 min of running to burn off the calories”; and the calorie label stands for “2630 kj per serving”.

Secondly, after reading the basic information of shrimp-flavored potato chips, participants were required to answer a series of questions to express their purchase intention and attitudes toward this product. Participants’ purchase intentions were rated by a 7-point scale (1 = definitely would not buy, and 7 = definitely would buy) (Huang et al. 2021). Thirdly, participants were asked to express how they felt about buying and eating this potato chips by three 7-point bipolar scales (not guilt-ridden/guilt-ridden, not ashamed/ashamed, and not remorseful/remorseful). These items were combined to form a scale for guilt (α = 0.928) (Elder and Mohr 2020). Finally, participants were informed to choose which kind of exercise they would prefer to burn calories, including walking slowly or running fast [6]. Through the investigation on the preference of consumers’ exercise, we can know which exercise has the most significant influence on the consumers, so as to determine the utility of different types of exercises to the consumers by means of data testing.

#### 3.1.3. Results

**Figure 4:**
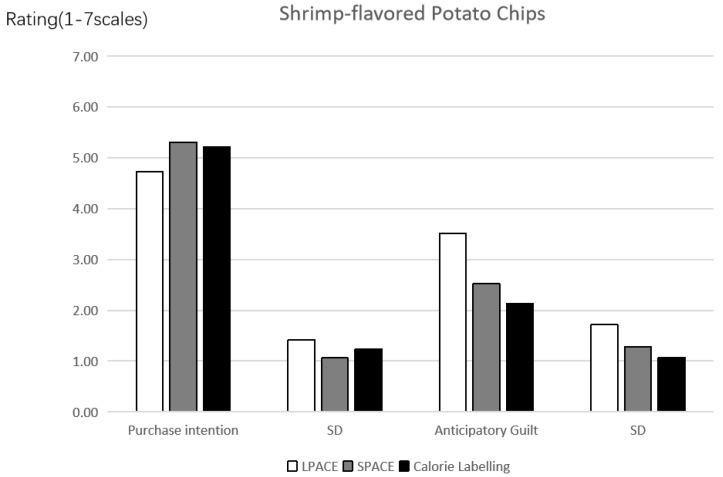
The effect of labels on purchase intention and anticipatory guilt.

Purchase intention. The experimental results of this study were depicted in Figure 4. A one-way analysis of variance (ANOVA) revealed the effects of different labels on the purchase intention of consumers for unhealthy food (*F*(2, 213) = 4.519, *p* = 0.012, *partial* *η*^2^ = 0.041). *F* represents the results of F-test on the purchase intention of consumers for unhealthy food in the three groups. It is shown that the three groups have significant differences without the same normal distribution in terms of purchase intention of consumers for unhealthy food. Partial *η*^2^ is the effect size of this experiment. If the partial *η*^2^ exceeds 0.01, it indicates that the independent variable has a small effect size on the dependent variable. If the partial *η*^2^ exceeds 0.06, it indicates that the effect size is medium. If the partial *η*^2^ exceeds 0.14, it indicates that the effect size is large. In addition, we also conducted power analysis on the experiment to measure whether the sample size of the experiment was sufficient. In this experiment, 1 − *β* was expressed as 0.766. This indicates that the sample size of the experiment is relatively sufficient.

The mean values of LPACE and other two groups were further analyzed by the t-test. The results show that consumers have lower purchase intention when the LPACE (vs. SPACE and calories label) is labeled on the front-of-pack of shrimp-flavored potato chips (M_LPACE_ = 4.73, SD = 1.424 vs. M_SPACE_ = 5.31, SD = 1.059, *p* = 0.005, and M_Calories_ = 5.22, SD = 1.236, *p* = 0.019); however, consumers’ purchase intention have no significant difference between the SPACE and calories label (*p* > 0.6); the average value of LPACE is significantly lower than that of other two groups, and the difference is proved to be significant by the t-test. Therefore, the Hypothesis 1 was verified to be valid.

Anticipatory guilt. The data analysis method is the same as the above mentioned. A one-way ANOVA revealed the effects of different labels on the anticipatory guilt (*F*(2, 213) = 18.883, *p* < 0.001, *partial* *η*^2^ = 0.151, and 1 − *β* = 1.0). It is found that consumers have higher anticipatory guilt when the LPACE of shrimp-flavored potato chips is used (vs. SPACE and calories label) (M_LPACE_ = 3.51, SD = 1.719 vs. M_SPACE_ = 2.52, SD = 1.275, *p* < 0.001, and M_Calories_ = 2.13, SD = 1.074, *p* < 0.001); however, consumers’ anticipatory guilt has no significant difference between the SPACE and calories label (*p* > 0.09).

**Figure 5:**
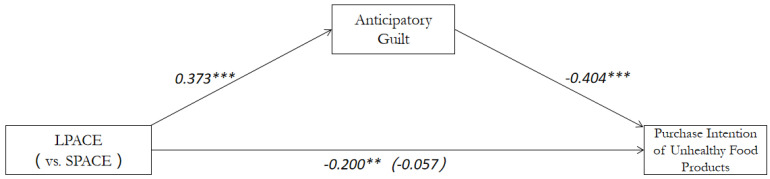
Study1: Mediating effect of anticipatory guilt between the labels and purchase intention. Note: Numbers indicate beta values, and Numbers indicate the main effect after adding the mediator variable. ** *p* < 0.01; *** *p* < 0.001.

Mediation analysis. As can be seen from the above analysis of purchase intention and anticipatory guilt, there is no significant difference between the SPACE and calories label. Therefore, these three labels were coded as LPACE = 1, and SPACE or calories label = 0. The anticipatory guilt was then taken as the mediator of the effect of labels on the purchase intention. In addition, the analysis of mediating effect of the model was carried out strictly according to the behavioral paradigm [69]. To be specific, a bootstrap analysis with 5000 samples was conducted by the Model 4 (it has one independent variable, one dependent variable and one mediation variable, and the mediation model using the anticipatory guilt is significant for the purchase intention of consumers for the unhealthy food (indirect effect = −0.3848, SE = 0.1152, and 95% CI = −0.6405~−0.1877) [70]. The interval range of CI does not contain 0, indicating that the mediating effect is significant [64,65,66,67]. Through the further analysis of regression coefficient, the mediation effect was also verified again. It is found that all the paths are significant (all *p* < 0.01) and the direction is consistent with the expectation (Figure 5). Thus, the Hypothesis 2 was also proven to be valid. At the same time, the proportion of direct effect (−0.1537) in the total effect (−0.5386) was also detected, and the proportion of direct effect was 28.57%. This indicates that the anticipatory guilt as a mediating variable can explain most of the mediating effect, but there are some other variables that can also influence the mediating effect of this model.

Exercise preference. The results show that a total of 133 participants (61.6%) prefer slow walking to burn calories, while 83 participants (38.4%) would want to choose fast running as a form of calorie-burning exercise. Through the main effect analysis, it is found that the consumers who prefer walk slowly are less likely to purchase the unhealthy food than those who choose fast running (M_walk_ = 4.98, SD = 1.337 vs. M_run_ = 5.27, SD = 1.127), but the difference isn’t significant (*p* > 0.1). In the further analysis, the exercise preference was taken as a moderating variable to evaluate the effect of labels on the purchase intention of consumers for the unhealthy food with different exercise preferences. The results show that there is no interaction between the exercise preference and label type (*p* > 0.7); at the same time, after controlling the exercise preference, the main effect of label form is still significant (*p* = 0.044). Specifically, we first consider the group whose sports preference is fast running, and find that LPACE has a significantly higher negative impact on purchase intention than SPACE (M_LPACE_ = 4.76, SD = 1.300 vs. M_SPACE_ = 5.44, SD = 0.943, *p* = 0.034). Then, we consider the group whose exercise preference is slow walking, and find that LPACE and SPACE have no significant negative impact on purchase intention, but LPACE’s purchase intention is lower (M_LPACE_ = 4.72, SD = 1.473 vs. M_SPACE_ = 5.10, SD = 1.205, *p* = 0.210), and the decrease in significance here may be the result of sample size reduction caused by grouping of subjects. It can be found that regardless of the form of exercise, LPACE makes consumers have a lower purchase intention for unhealthy food. To sum up, the experimental results indicated that the LPACE and SPACE have different inhibitory effects on the purchase intention of consumers for the unhealthy food with different exercise preferences, and the effects are stable in this study.

### 3.2. Study 2: Re-Examination of Main and Mediating Effects

In Study 2, combined with the consumption scenario of chocolate, Hypotheses 1 and 2 were proved to be valid. The intergroup design of LPACE, SPACE and calories label was used for the comparison. However, Study 2 is different from Study 1 in several aspects. On one hand, the dependent variable in Study 2 increased the measurement of consumers’ recommendation intention, which proved the difference in consumers’ purchase intention for food from multiple dimensions. On the other hand, the potential mediation mechanism of emotions (except for the guilt) was eliminated.

#### 3.2.1. Methodology

A total of 250 high-reliability respondents were recruited from the Credamo online platform and were randomly assigned to one of the following three experimental groups, i.e., LPACE, SPACE and calories label. After checking whether there was a “Z” law or those who failed to recall the labels, 20 samples were excluded, and 230 valid questionnaires (Table 2) were collected, including 76 respondents in the LPACE group, 77 respondents in the SPACE group, and 77 respondents in the calories label group.

#### 3.2.2. Procedure and Materials

**Figure 6:**
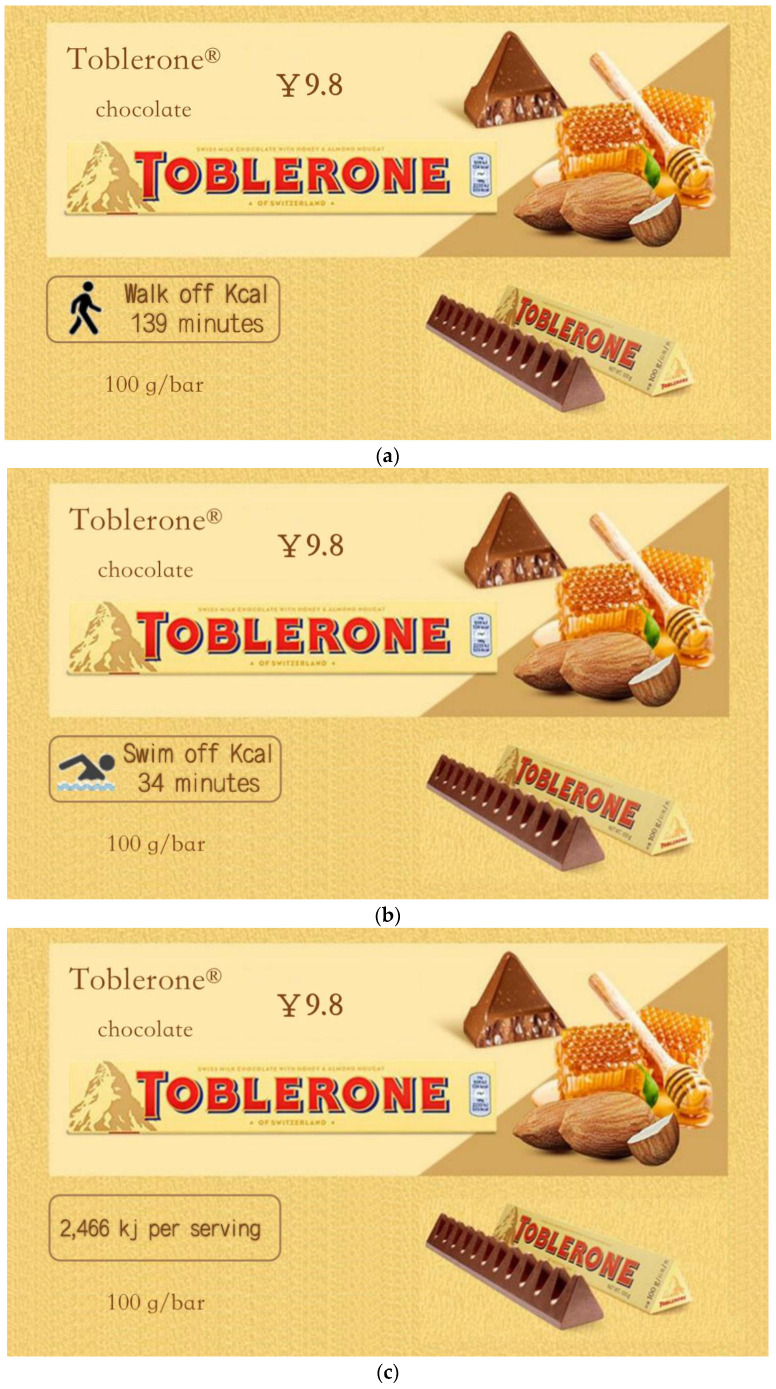
Stimuli of study 2. Note: (**a**) the LPACE group, (**b**) the SPACE group, and (**c**) the calories label group.

The experimental procedure of study 2 was the same as that of study 1. Participants were asked to know about the general information of Toblerone, and accordingly assess and rate it. The background knowledge of the experiment is that one food company is going to release a novel fictional Toblerone of “Toblerone” (100 g/bar, 9.8 yuan). The LPACE, SPACE and calories label were used as the product introduction, of which LPACE stands for “it needs 139 min of walking to burn off the calories“; SPACE represents “it needs 34 min of swimming to burn off the calories“; and the calorie label is “2466 kj per serving” (Figure 6). In addition, participants were randomly assigned to one of these three groups.

In this Toblerone experiment, the purchase intention of participants for product attributes was firstly evaluated, followed by the anticipatory guilt (α = 0.879) and recommendation intention. The item of recommendation intention for this Toblerone is “I recommend this Toblerone to someone who wants to buy chocolate products” (1 = not at all, and 7 = very much so). This measurement items were cited from Lee et al. (2010). In addition, consumers were required to select which type of exercise they would wish to burn calories, such as walking slowly or swimming. Positive and negative effects of participants were also scored by a 7-point scale in the end. Items “enthusiast”, “interested”, and “excited” were chose as positive effect items (α = 0.892), and items “upset”, “offended”, and “irritable” were selected as negative effect ones (α = 0.895) (Huang et al., 2021). By excluding emotions other than guilt, the robustness of the mediation mechanism proposed in this paper was ensured.

#### 3.2.3. Results

**Figure 7:**
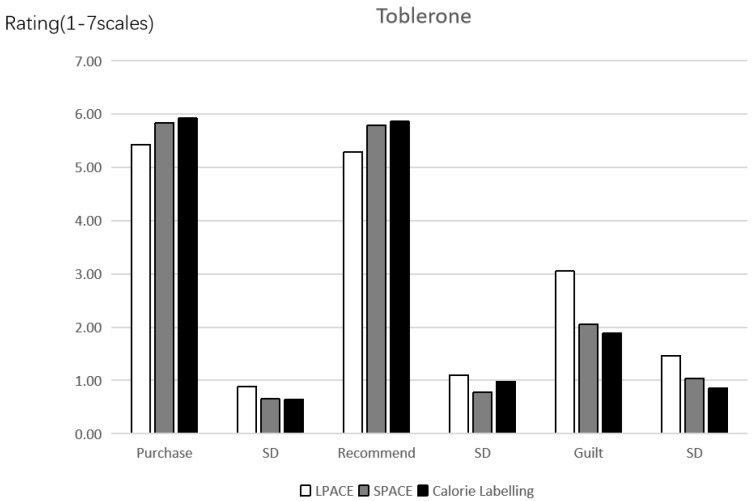
Effects of labels on the purchase intention, recommendation intention and anticipatory guilt of consumers.

Purchase intention. The data analysis method of the results of study 2 was the same as that of study 1. Results of this study were depicted in Figure 7. A one-way ANOVA revealed the influences of different labels on the purchase intention of consumers for the unhealthy food (*F*(2, 227) = 9.731, *p* < 0.001, *partial* *η*^2^ = 0.079, and 1 − *β* = 0.982). The results indicate that consumers possess lower purchase intention when the LPACE (vs. SPACE and calories label) is adopted as the front-of-pack of Toblerone (M_LPACE_ = 5.43, SD = 0.884 vs. M_SPACE_ = 5.84, SD = 0.650, *p* = 0.001, and M_Calories_ = 5.92, SD = 0.644, *p* < 0.001). However, the consumers’ purchase intention shows no significant difference between the SPACE and calories label (*p* > 0.5). Hence, the Hypothesis 1 was verified to be valid again.

Recommendation intention. A one-way ANOVA reflected the effects of different labels on the recommendation intention of consumers for the unhealthy food (*F*(2, 227) = 7.900, *p* < 0.001, *partial* *η*^2^ = 0.065, and 1 − *β* = 0.952). It is shown that consumers present lower recommendation intention when the LPACE (vs. SPACE and calories label) is chose as the front-of-pack (M_LPACE_ = 5.29, SD = 1.105 vs. M_SPACE_ = 5.79, SD = 0.784, *p* = 0.001, and M_Calories_ = 5.86, SD = 0.983, *p* < 0.001). However, the consumers’ purchase intention has no significant difference between the SPACE and calories label (*p* > 0.6). Therefore, the Hypothesis 1 was proven to be valid from another dimension.

Anticipatory guilt. A one-way ANOVA indicated the effects of different labels on the anticipatory guilt (*F*(2, 227) = 22.845, *p* < 0.001, *partial* *η*^2^ = 0.168, and 1 − *β* = 1.0). Consumers perceived higher anticipatory guilt when the LPACE was adopted (vs. SPACE and calories label) (M_LPACE_ = 3.05, SD = 1.468 vs. M_SPACE_ = 2.05, SD = 1.033, *p* < 0.001, and M_Calories_ = 1.89, SD = 0.851, *p* < 0.001). However, the anticipatory guilt has no significant difference between SPACE and calories label (*p* > 0.3).

Mediation analysis. In Study 1, the mediation model of this study was analyzed. It is found that the mediation model using the anticipatory guilt is significant (indirect effect = −0.2045, SE = 0.0633, 95% CI = −0.3429~−0.0946). At the same time, the proportion of direct effect (−0.2444) in the total effect (−0.4486) was also detected, and the proportion of direct effect was 54.48%. This indicates that the anticipatory guilt as a mediating variable can explain most of the mediating effect. As result, the Hypothesis 2 was proven again.

Exercise preference. A total of 172 participants (74.8%) preferred slow walking to burn calories, while 58 (25.2%) selected swimming as a form of calorie-burning exercise. Through the main effect analysis, it is found that the consumers who prefer to walk slowly are significantly much more impossible to purchase the unhealthy food than those who prefer to swim (M_walk_ = 5.66, SD = 0.752 vs. M_swimming_ = 5.97, SD = 0.748, *p* = 0.025), which is the same as that in study 1. It is suggested that consumers without strenuous exercise are more sensitive to the three calorie labels.

In further analysis, the exercise preference was adopted as a moderating variable to explore the effect of label type on the purchase intention. The results show that there is an interaction between the exercise preference and label type (*p* = 0.025); meanwhile, after controlling the exercise preference, the main effect of label form is still significant (*p* < 0.001). Therefore, in Study 2, consumers’ exercise preference had an influence on their purchase intention for the unhealthy food, and also moderated the main effect of different labels on their purchase intention, but it did not affect the significance of main effect in this paper. In other words, this main effect can exist in people with different exercise preferences.

Control variable. The mediating effect of emotion other than the guilt was also excluded in this study. Both positive and negative emotions had no significant difference among three labels groups (*F_p_*(2, 227) = 1.945, *p* > 0.1; *F_n_*(2, 227) = 2.156, *p* > 0.1). It is suggested that other major positive and negative emotions other than the guilt have not significant differences in the three groups, thus ruling out the possible influence of other emotions except for the guilt on the purchase intention of consumers for the unhealthy food.

### 3.3. Study 3: Re-Examination of Main and Mediating Effects

The intergroup design of LPACE + calories label and SPACE + calories label for comparison are used in this experiment. This is to better simulate what happens in the real world when the PACE label is presented alongside the calorie label. This also examine whether when calorie information appeared alongside the PACE label, the negative effects of LPACE on purchase intention for unhealthy food will still be there. In addition, we also verified that consumer demographics such as gender, age, income, education and BMI did not affect the main effect of LPACE on purchase intention of unhealthy foods.

#### 3.3.1. Methodology

The logic of Study 3 is similar to Study 1 and Study 2. In this research, 300 high-reliability respondents were randomly recruited from the Credamo and assigned to one of the following two experimental groups: LPACE + calories label and SPACE + calories label. After checking whether there is a “Z” law or those fail to recall the labels. 27 samples were excluded and 273 valid questionnaires were collected, including 134 respondents in the LPACE + calories label group, and 139 respondents in the SPACE + calories label group.

#### 3.3.2. Procedure and Materials

**Figure 8:**
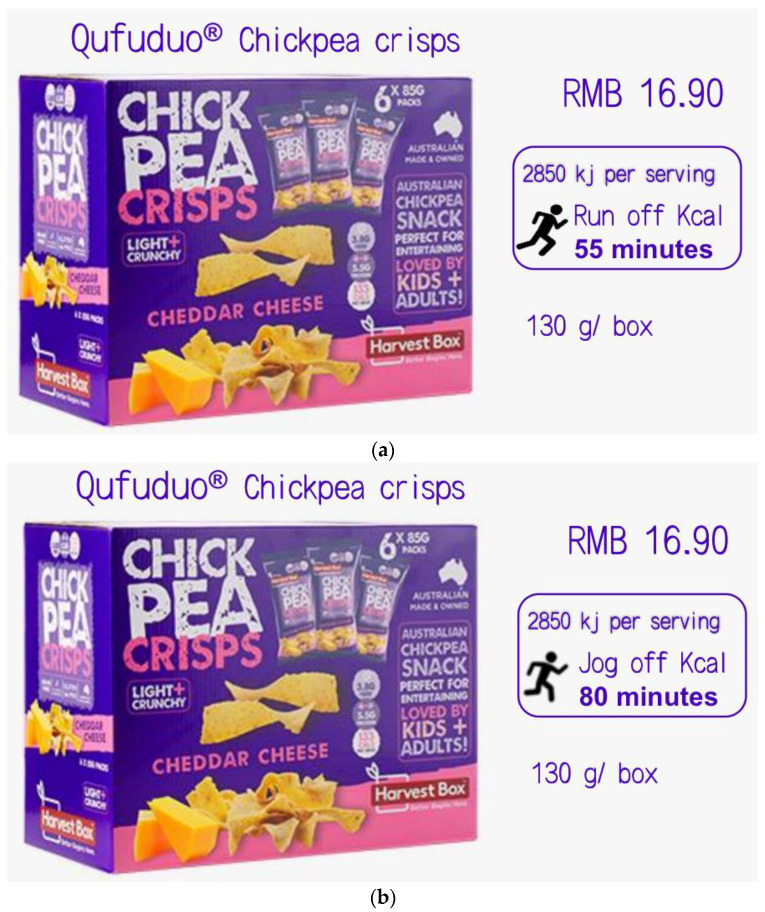
Stimuli of study 3. Note: (**a**) the LPACE + calories label group and (**b**) the SPACE + calories label group.

Participants were randomly assigned to one of the two groups. First of all, participants should read the basic information of chickpea crisps, and accordingly evaluate it. The background information of the product in this experiment is that a food company is about to launch a new fictitious chickpea crisps named Good Life Finute (130 g/bag, 16.9 yuan). The LPACE + calories label or SPACE + calories label was attached to the product introduction in the two groups. The specific details are as follows (Figure 8): LPACE means “it needs 80 min of jogging to burn off the calories”; SPACE refers to “it needs 55 min of running to burn off the calories”; and the calorie label stands for “2850 kj per serving”.

Secondly, after reading the basic information, participants were required to answer a series of questions. In this experiment, the purchase intention of participants for product attributes was firstly evaluated, followed by the anticipatory guilt (α = 0.903) and recommendation intention. Finally, participants filled in their demographic characteristics, such as sex, age, income, education, BMI.

#### 3.3.3. Results

**Figure 9:**
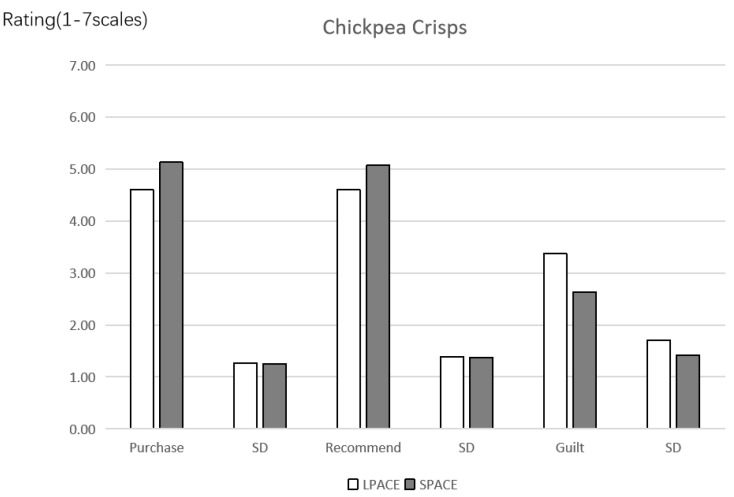
The effect of labels on purchase intention, recommendation intention and anticipatory guilt.

Purchase intention. The experimental results of this study were depicted in Figure 9. A one-way ANOVA revealed the effects of different labels on the purchase intention of consumers for unhealthy food (*F*(1, 271) = 12.251, *p* = 0.001, *partial* *η*^2^ = 0.043, and 1 − *β* = 0.937). The results show that consumers have lower purchase intention when the LPACE + calories label (vs. SPACE + calories label) is labeled on the front-of-pack of unhealthy food (M_LPACE_ = 4.60, SD = 1.263 vs. M_SPACE_ = 5.13, SD = 1.250). Therefore, the Hypothesis 1 was verified to be valid.

Recommendation intention. A one-way ANOVA reflected the effects of different labels on the recommendation intention of consumers for the unhealthy food (*F*(1, 271) = 8.090, *p* = 0.005, *partial* *η*^2^ = 0.029, and 1 − *β* = 0.809). It is shown that consumers present lower recommendation intention when the LPACE + calories label (vs. SPACE + calories label) is chose as the front-of-pack (M_LPACE_ = 4.60, SD = 1.388 vs. M_SPACE_ = 5.07, SD = 1.371).

Anticipatory guilt. The data analysis method is the same as the above mentioned. A one-way ANOVA revealed the effects of different labels on the anticipatory guilt (*F*(1, 271) = 15.055, *p <* 0.001, *partial* *η*^2^ = 0.053, and 1 − *β* = 0.972). It is found that consumers have higher anticipatory guilt when the LPACE + calories label of unhealthy food is used (vs. SPACE + calories label) (M_LPACE_ = 3.37, SD = 1.711 vs. M_SPACE_ = 2.63, SD = 1.420).

Mediation analysis. Two labels were coded as LPACE + calories label = 1, and SPACE + calories label = 0. The anticipatory guilt was then taken as the mediator of the effect of labels on the purchase intention. In addition, a bootstrap analysis with 5000 samples was conducted by the Model 4 (indirect effect = −0.2465, SE = 0.0740, and 95% CI = −0.4017~−0.1120). The interval range of CI does not contain 0, indicating that the mediating effect is significant. Thus, the Hypothesis 2 was also proven to be valid. At the same time, the proportion of direct effect (−0.2860) in the total effect (−0.5325) was also detected, and the proportion of direct effect was 53.71%. This suggests that anticipatory guilt is a partial mediator in the model.

Demographic characteristics. The experiment needs to exclude the effect of demographic characteristics on PACE label. It is found that the main effect of gender on the purchase intention is not significant (*F*(1, 269) = 4.45, *p* = 0.036), and there is no interaction effect with the PACE label (*p* > 0.5). To be specific, compared with male (M = 5.07), female subjects (M = 4.72) have lower purchase intention for unhealthy food, but gender does not have a moderating effect on the effect of LPACE, indicating that the effect of PACE label is relatively stable in different gender groups. The education was divided into three categories: college/high school and below, undergraduate, master’s degree and above. The results show that the education has no main effect on the purchase intention (*F*(2, 267) = 4.02, *p* > 0.05), and no interaction effect with the PACE label (*p* > 0.5). The income was divided into less than 2000, 2001–4000, 4001–6000, 6000 and above. The results show that the main effect of income on the purchase intention is significant (*F*(3, 204) = 5.764, *p* = 0.001), but it has no significant interaction effect with the PACE label (*p* > 0.1). Therefore, although different income groups have different purchasing intentions for unhealthy food, the effect of LPACE label is stable in different income groups. The age was divided into four kinds: less than 20 years old, 21–30 years old, 31–40 years old, 41 years old and above, and the purchase intention was regressed. The results show that the age has no main effect on the purchase intention (*F*(3, 258) = 1.211, *p* > 0.1), and no interaction effect with the PACE label (*p* > 0.5). The BMI was divided into three categories: ≤18.4, 18.5–23.9, ≥24.0. The results show that the BMI has no main effect on the purchase intention (*F*(2, 270) = 0.06, *p* > 0.5), and no interaction effect with the PACE label (*p* > 0.5). Therefore, the moderating or interactive effect of subjects’ demographic characteristics on the purchase intention for ugly food with PACE label was excluded.

### 3.4. Study 4: Examination of Moderation effects

In Study 4, based on the consumption scenario of cookies, the boundary condition of main effect (Hypothesis 3) was verified. It is predicted that when consumers have high future self-continuity (abbreviated as HIGH), compared with the short time PACE, the longtime PACE has a stronger influence on reducing the consumers’ purchase intention for unhealthy food products; however, when consumers are in low future self-continuity (abbreviated as LOW), there is no significant difference in the effect between the short time and longtime PACEs. The floodlight method was used to verify the important boundary effect of consumers’ future self-continuity. In addition, the dependent variable increased the measurement of consumers’ perceived value. Therefore, the 2 × 2 mixed design was used, including two intergroup labels (LPACE vs. SPACE) and two intergroup future self-continuities (HIGH vs. LOW). At the same time, the potential mediation mechanism involved in food choices was eliminated.

#### 3.4.1. Methodology

In the experiment, 260 high-reliability respondents were recruited from the Credamo online platform and randomly assigned to one of the following four experimental groups: HIGH-LPACE, LOW-LPACE, HIGH-SPACE and LOW-SPACE. After checking whether there is a “Z” law among them or those who failed to recall the labels, 28 samples were excluded and 232 valid questionnaires were collected, including 58 respondents in the HIGH-LPACE group, 55 respondents in the LOW-LPACE group, 60 respondents in the HIGH-SPACE group and 59 respondents in the LOW-SPACE group.

#### 3.4.2. Procedure and Materials

**Figure 10:**
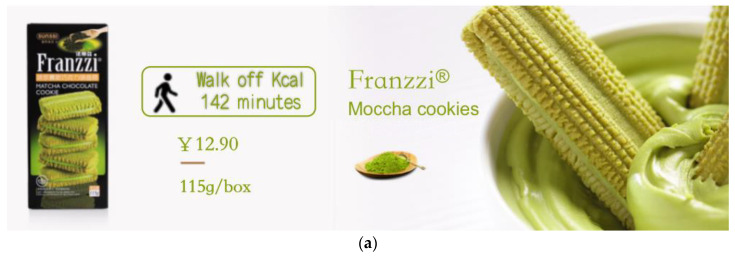

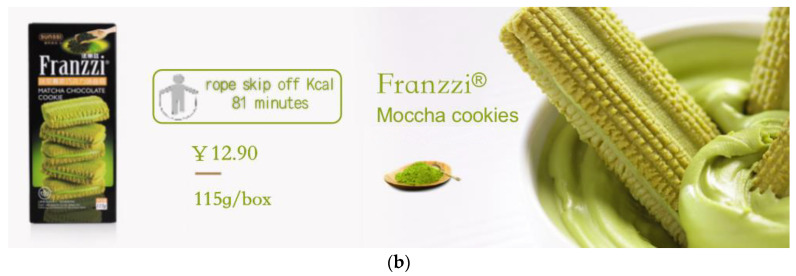
Stimuli of study 4. Note: (**a**) the LPACE group and (**b**) the SPACE group.

Participants were randomly assigned to one of four groups “HIGH-LPACE, LOW-LPACE, HIGH-SPACE and LOW-SPACE”. They were required to have a knowledge of Moccha cookie, and accordingly estimate and score it. The background information of this experiment is that a food company is about to develop a new Moccha cookie of “Franzzi” (115 g/box, 12.9 yuan). The LPACE and SPACE were adopted as the labels of “Franzzi”, of which LPACE refers to “it needs 142 min of walking to burn off the calories”; SPACE stands for “it needs 81 min of rope skipping to burn off the calories” (Figure 10).

The manipulation of future self-continuity was carried out, and writing tasks were used in this study. Participants were asked to talk or write to manipulate the future self-continuity [71,72]. In the HIGH group, participants were required to imagine and describe 2 similarities between their present self and future self 5 years later in detail, and explain why this similarity was maintained over 5 years (8–14 words). In the LOW group, participants were told to imagine and describe two specific ways in which their present self would be different from their future self 5 years from now, and to explain why this difference occurred 5 years from now (8–14 words).

In this Moccha cookie experiment, the purchase intention of participants for unhealthy product was firstly evaluated, and the anticipatory guilt (α = 0.910) and perceived value were then assessed. The items of perceived value for this cookie are “I think this cookie was well made”, “I think the quality of this cookie is acceptable”, “I think this cookie was worth the money”, and ”I think this cookie is economical” (1 = not at all, and 7 = very much so) (α = 0.811). This measurement items were adapted from Sweeney and Soutar (2001).

**Figure 11:**
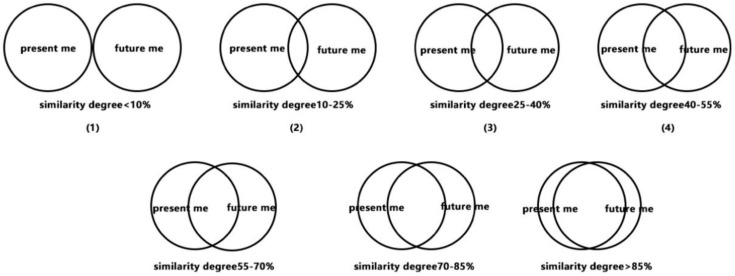
Questionnaire of the future self-continuity testing.

Whether high and low future self-continuities between different groups were manipulated was measured successfully later. The method for measuring the future self-continuity by allowing subjects to make schema choices has been adopted by many empirical studies [73,74]. Specifically, 7 groups of circles were shown in Figure 11. In each group, the circles on the left and right represent the present self and future self, respectively. The degree of overlap between the two circles represents the continuous relationship between the present self and the future self. The greater the degree of overlap, the stronger the continuity of the future self.

Participants were asked to choose which form of exercise they would prefer next, and then answer a question “I think that the decision to purchase upcycled food is ?” (1 = very unimportant decision, and 7 = very important decision) [75]. The purity of the main effects in this study was improved by excluding the influence of consumers’ involvement in food choices.

#### 3.4.3. Results

**Figure 12:**
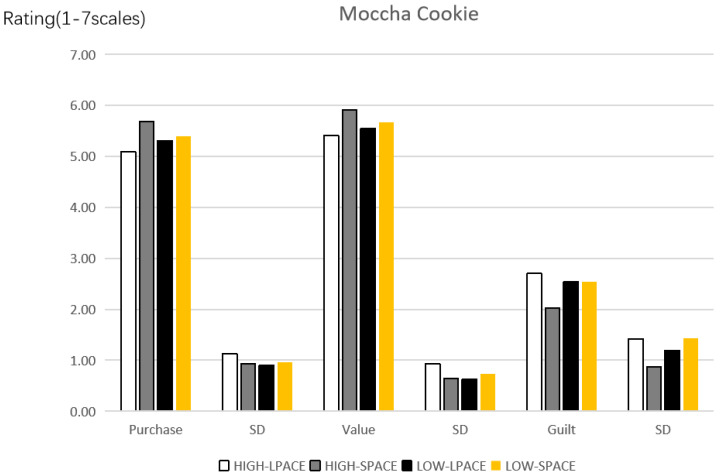
Effect of labels on the purchase intention, perceived value and anticipatory guilt of consumers.

Manipulation check. The experimental results of this study were depicted in Figure 12. Whether there was any difference in the degree of future self-continuity between the HIGH and LOW groups after manipulation was compared. The results show that the degree of future self-continuity in the HIGH group is much higher than that in the LOW group, and the difference is significant (M_HIGH_ = 5.50, SD = 0.884 vs. M_LOW_ = 4.34, SD = 0.958, *p* < 0.001), indicating that the manipulation of future self-continuity is successful.

Purchase intention. Results of Study 4 were shown in Figure 10. Through a two-way ANOVA, a significant interaction between the labels (LPACE vs. SPACE) and the future self-continuities (HIGH vs. LOW) was revealed (*F*(3, 228) = 3.705, *p* = 0.012, *partial* *η*^2^ = 0.046, and 1 − *β* = 0.801). In the context of high future self-continuity, consumers had lower purchase intention when the LPACE was selected (vs. SPACE) (M_LPACE_ = 5.09, SD = 1.128 vs. M_SPACE_ = 5.68, SD = 0.930, *p* = 0.002). However, in the context of low future self-continuity, consumers’ purchase intention had no significant difference (M_LPACE_ = 5.31, SD = 0.900 vs. M_SPACE_ = 5.39, SD = 0.965, *p* > 0.6). Therefore, Hypotheses 1 and 3 were verified to be valid.

Perceived value. Through a two-way ANOVA, a significant interaction between the label (LPACE vs. SPACE) and the future self-continuity (HIGH vs. LOW) was revealed (*F*(3, 228) = 4.026, *p* = 0.003, *partial* *η*^2^ = 0.061, and 1 − *β* = 0.906) on the perceived value. In the context of high future self-continuity, consumers had lower perceived value when the LPACE was used (vs. SPACE) (M_LPACE_ = 5.41, SD = 0.928 vs. M_SPACE_ = 5.91, SD = 0.642, *p* = 0.001). However, in terms of low future self-continuity, consumers’ perceived value had no significant difference in it (M_LPACE_ = 5.55, SD = 0.630 vs. M_SPACE_ = 5.67, SD = 0.733, *p* > 0.3).

Anticipatory guilt. Through a two-way ANOVA, a significant interaction between the label (LPACE vs. SPACE) and the future self-continuity (HIGH vs. LOW) was reflected on the anticipatory guilt (*F*(3, 228) = 4.245, *p* = 0.021, *partial* *η*^2^ = 0.042, and 1 − *β* = 0.747). In the context of high future self-continuity, consumers had higher anticipatory guilt when the LPACE was adopted (vs. SPACE) (M_LPACE_ = 2.70, SD = 1.424 vs. M_SPACE_ = 2.02, SD = 0.874, *p* = 0.002). However, for low future self-continuity, consumers’ anticipatory guilt also had no significant difference in it (M_LPACE_ = 2.54, SD = 1.194 vs. M_SPACE_ = 2.54, SD = 1.426, *p* > 0.9).

Mediation analysis. A moderated mediation analysis was implemented to investigate the predicted relationship. To be more specific, the labels were used as the predictor variables, and the future self-continuity and anticipatory guilt were adopted as the mediators. The bootstrap analysis with 5000 samples using the Model 7 (moderated mediation) indicates that the full model is significant (R^2^ = 0.2787, and *p* < 0.0001). In addition, the *p* represents that the model is significant, and R^2^ means that the model’s fitting degree is good. In further analysis, the anticipatory guilt mediated the effects of labels on the purchase intention for high future self-continuity (indirect effect = −0.2683, SE = 0.1001, 95% CI = −0.4870~−0.0928), rather than low future self-continuity (indirect effect = 0.0012, SE = 0.0981, 95% CI = −0.1938~0.1957). At the same time, the proportion of direct effect (−0.2079) in the total effect (−0.4774) was also detected, and the proportion of direct effect was 43.55%. This indicates that the anticipatory guilt as a mediating variable can explain most of the mediating effect. Similarly, after replacing the purchase intention with the perceived value, the anticipatory guilt also mediated the effects of labels on the perceived value for high future self-continuity (indirect effect = −0.1668, SE = 0.0645, 95% CI = −0.3093~−0.0566), instead of low future self-continuity (indirect effect = 0.0007, SE = 0.617, 95% CI = −0.1184~0.1275). Hence, Hypothesis 2 was verified to be valid again.

Future self-continuity. The moderating effect of consumers’ future self-continuity for unhealthy food was further examined in this section. Since the moderator was a cardinal variable, the Johnson–Neyman floodlight analysis technique was used to examine the constraining effect of LPACE on the purchase intention of consumers for unhealthy food across the entire range of consumers’ future self-continuity [76]. The PROCESS Model 1 (Hayes 2017) representing the causality test between the independent and dependent variables under different moderating variables was adopted to run the floodlight analysis, of which PROCESS is a tool for path analysis-based moderation and Model 1 contains moderator, independent and dependent variables. With 95% CI, the constraining effect of LPACE on the purchase intention of consumers for unhealthy food is significant for the future self-continuity, which is more than 4.5899 (71.5517% of the participants). Moreover, the effect is out of operation (*p* > 0.05) for the future self-continuity, which is below 4.5899 (28.4483% of the participants). The boundary value of future self-continuity for studying the Hypothesis 3 was further determined, and the floodlight method provided a future self-continuity threshold for subsequent related research.

Control variable. In order to exclude the mediating alternative explanation of consumers’ involvement in food choices. The mediating effect involved in food choices without significant difference among these four groups was also excluded (*F*(3, 228) = 1.262, and *p* > 0.2). The results showed that there was no significant difference in the involvement in food choices of the four groups, indicating that there was no significant difference in the influence of LPACE label and SPACE label on the involvement in food choices of consumers. Thus, the possible influence involved in food choices on the purchase intention of consumers for unhealthy food was ruled out.

## 4. General Discussion

In this study, the three hypotheses are verified through four scenario experiments. It has proved that compared with the short time PACE and calorie labels, the longtime PACE labels have more inhibitory influence on consumers’ purchase intention for unhealthy food. At the same time, the role of anticipatory guilt in the above inhibition effect is further analyzed. In addition, the future self-continuity of consumers is used as the moderating boundary of this inhibition effect. The possibly potential effect of exercise preference on the inhibitory effect of PACE labels on the unhealthy food is also analyzed. Based on the results of this study, four important research conclusions and theoretical contribution are finally summarized and discussed in combination with the literature.

Firstly, it is proved that different forms of PACE have different inhibitory effects on the purchase intention of consumers for unhealthy food, which not only deepens the studies on different forms of PACE labels, but also resolves the contradictory problems about the main effects of PACE labels [30]. The reason why we proved this paradox is that the different forms of PACE labels are not distinguished [16,31]. It is also verified that long time PACE is more effective than calorie label, which is consistent with the results of previous studies [22,23]. At the same time, there is no significant difference in the inhibitory effect of short time PACE and calorie labels on the unhealthy food, which is also accordance with the results of previous studies, that is, there is no significant difference between the PACE and calorie labels [25]. In addition, the first study is to divide the PACE into long time and short time PACE labels, and to verify the causal relationship between the PACE labels and purchase intention for unhealthy food through a situational experiment. Yang et al. (2021) further provided a paradigm for subsequent PACE studies, and distinguished different types of PACE labels so as to evaluate their specific roles [6].

Secondly, the anticipatory guilt is introduced to explain the inhibitory effect of PACE labels, which complemented the gaps in the psychological mechanism in previous PACE labeling studies [36,48]. However, previous relative studies have focused on the promoting or inhibiting effects of food selection, but only briefly discussed the psychological mechanism without further causal analysis [27,77]. This study not only analyzed the theoretical source of inhibitory effect of long time PACE labels by combining time pressure and calorie risk [32,33], but also systematically explored the underlying mechanism of inhibitory effect by anticipating the negative emotion of guilt [35]. Furthermore, it provides a new perspective and deeper psychological insight to understand how to nudge consumers to form good and healthy consumption behaviors, which lead to a theoretical improvement of nudge theory in the field of food consumption essentially [11].

Thirdly, it is not limited to the traditional demographic characteristics as the boundary regulation of the PACE effect [50]. The perceptual factor of time dimension “future self-continuity” is adopted as the moderating variable of PACE labels effect, which is more conducive to understanding the influence of consumers’ deep personality on the PACE labels effect [29,54]. Although the influence of future self-continuity on consumers’ food choices is tentatively explored in previous studies [78], the future self-continuity is introduced to explore in the effects of PACE labels on non-healthy food choices for the first time in this study [57], as well as analyze the differences in the effects between different kinds of PACE labels. In addition, future self-continuity as a moderating variable provided a suitable applicable boundary for food enterprises to gain insight into consumers, and linked the effects of PACE labels on the concepts, such as consumers’ self-control [55,56].

Fourthly, it is found that consumers’ exercise preference may have a little negative effect on the purchase intention of consumers for unhealthy food under the influence of PACE labels. Several experiments have shown that consumers who prefer slow movements (such as walking) are more sensitive to the PACE than those who prefer fast movements (such as running, rope skipping, or swimming), leading to lower purchase intention for unhealthy food under the influence of PACE. It is reflected that in a certain type of consumer group, the inhibitory effect of long time and short time PACE labels on the purchase intention of consumers for unhealthy food has been strengthened. In further study, the exercise preference can be used as an important moderating variable in the PACE research. At the same time, according to the existing literature [6], the effect of PACE labels from the perspective of exercise preference has not been studied and analyzed. In addition, the experimental results also prove that the exercise preference does not affect the strong inhibitory effect of long time PACE labels on the consumers’ purchase intention for unhealthy food, indicating that the constraining effects of long time PACE labels on the purchase intention for unhealthy food are applicable to different consumer groups.

### 4.1. Practical Implication

The results of this study can also provide some practical implications for the government, health organizations and food enterprises. As mentioned before, a large number of current global health problems are caused by unhealthy food habits [1,3], which results in heavy financial burden on governments and medical care [5,7]. With visual calorie, PACE labels can be used as a nudge tool to effectively encourage consumers to avoid the unhealthy food [11,12], and help address a number of problems associated with unhealthy food [13], which is encouraged by the government and health organizations. However, in order to better reflect the idea of nudge, that is, to provide better intervention effect at lower cost [9,10], it is suggested that because it has a stronger guiding role for consumers in healthy consumption, the food should be labeled with a long time PACE label rather than a short time PACE label in practical application, which has been demonstrated repeatedly in previous studies [6], and achieves a significant inhibitory effect on the unhealthy food. For the government and health organizations, simply replacing calorie and short time PACE labels with long time PACE labels at low cost can have a better effect, which is a double result with half the effort.

At the same time, companies that produce healthy foods should actively participate in the movement to label calories through the PACE labels. This is because it is likely to cause consumers to spillover from unhealthy food and thus increase the possibility of purchasing healthy food. In addition, snack makers may have little incentive to push nudges such as PACE labels, which can decrease the possibility of purchasing unhealthy foods with high calories [6]. To promote the consumption of healthy food, governments and health organizations should develop industry regulations that require such enterprises to attach the PACE labels, especially form of long time PACE. All in all, PACE, as a practical boosting tool, brings unlimited possibilities for promoting the consumption of healthy food and better managing the food calorie labeling system, which provides richer practical significance for governments, health organizations and food companies.

### 4.2. Limitations and Future Research Directions

Limitations in this study are summarized and the future research directions are also put forward accordingly as follows:

First of all, the situational experiments verify the causal relationship between the variables and hypothesis proposed in this study. However, there are also methodological shortcomings. For example, although a variety of dependent variable measurements are used for respondents by the situational experiment method, including the purchase intention, perceived value, word-of-mouth publicity, etc., there are some discrepancies with actual purchase behaviors. At the same time, although the scenario experiment simulates the food selection situation, there are more interference and influence factors in the real shopping environment. In order to make the experimental results cleaner, the situational experiment method often controls certain possible interference factors. As a result, the external validity of research results also needs to be added in this paper, which can better reflect the real consumers’ behavior and psychology. In the further study, field and selective experiments should be adopted to enhance the rationality of dependent variable measurement, and more real environmental factors should be added to strengthen the external validity of research results. At the same time, future research will also increase the sample size of the experiment and include a more diverse group of participants, so as to improve the universality of research conclusions for different consumer groups.

Secondly, due to the limitations of this study, the moderating effect of consumers’ future self-continuity, exercise preference, emotions, involvement in food choices and demographics on the inhibiting effect of long time PACE labels was discussed in this study. In the actual consumption scenario, to further expand the research breadth in the field of health consumption and food labeling, more consumer and product characteristics deserve further discussion, such as health goals of consumers, health degrees of food, taste, price and knowledge. Meanwhile, in the follow-up, more external environmental variables should be considered to build a theoretical model about the inhibitory effect of PACE labels on the food consumption through the structural equation modeling and other methods, so as to form a series of related research results, and enrich the practical significance of PACE labels as well. In addition, future research will examine the impact of PACE on consumers’ willingness to participate in physical activity after food selection, in order to more deeply evaluate the effects of LPACE.

Finally, this study has repeatedly verified that anticipatory guilt is the mediating variable of the negative effects of LPACE label on purchase intention for unhealthy food. However, through the mediation analysis of the experiment, it can be seen that there are some other variables that can also influence the mediating effect of this model. In future studies, we hope to introduce justification as another mediating interpretation mechanism. Justification means to use available reasons to rationalize choices or behaviors, which can resolve possible conflicts and feelings of guilt [79]. For example, Alba and Williams (2013) concluded in their review of hedonism research literature that when consumers can justify indulgence consumption, the proportion of hedonistic products and indulgence consumption will increase significantly [80]. To this end, we will further analyze anticipatory guilt and non-healthy food purchasing behavior of consumers under the influence of PACE labeling from the perspective of justification.

## 5. Conclusions

This study demonstrates that different PACE labels have different influence on unhealthy food consumption. PACE labels are divided as the long time and the short time PACE labels based on different sports in this study. Through four experiments, results show that compared with the short time PACE and calorie labels, the longtime PACE labels have more negative influence on consumers’ purchase intention for unhealthy food. Furthermore, the anticipatory guilt is adopted to explain the negative effect of PACE labels, the reason is that consumers are often prone to feeling guilty in the process of unhealthy food consumption. The perceptual factor of time dimension “future self-continuity” is adopted as the moderating variable of PACE labels effect, results show that individuals with high future self-continuity have higher self-control and take more consideration of future outcomes, they are reluctant to choose unhealthy food than others. Finally, it is found that consumers’ exercise preference may have a little negative effect on the purchase intention of consumers for unhealthy food under the influence of PACE labels.

## Figures and Tables

**Table 1:** General idea of experimental design.

Study	Hypothesis	Grouping	Major Variables	Stimulant	Movement
1	H1 & H2	Intergroups: label (LPACE vs. SPACE vs. calories)	Purchase intention, Exercise preference	potato chips	Walk vs. running
2	H1 & H2	Intergroup: label (LPACE vs. SPACE vs. calories)	Recommendation intention, Emotions (except guilt)	chocolate	Walk vs. swimming
3	H1 & H2	Intergroup: label (LPACE + calories vs. SPACE + calories)	Purchase intention, Recommendation intention, Demographic	Chickpea crisps	Jogging vs. running
4	H1 & H2 & H3	Intergroup: 2 labels (LPACE vs. SPACE) × 2 future self-continuity (high vs. Low)	Perceived value, Involvement in food choices	cookies	Walk vs. rope skipping

**Table 2:** Socio-demographics of consumers in Study 1–3.

Socio-Demographic Indicators		Study 1	Study 2	Study 3	Study 4
Variable	Definitions	Percentage	Percentage	Percentage	Percentage
Gender	Male	43.5%	57.0%	42.9%	51.7%
	Female	56.5%	43.0%	57.1%	48.3%
Age	≤20 years old	3.2%	2.6%	8.1%	2.6%
	21–30 years old	67.6%	71.7%	53.5%	67.2%
	31–40 years old	22.2%	24.3%	29.7%	27.6%
	≥41 years old	6.9%	1.3%	8.8%	2.6%
Education	Senior high school and below	4.2%	1.7%	7.7%	2.2%
	junior college	17.1%	11.7%	11.0%	12.5%
	bachelor’s degree	70.4%	76.1%	69.6%	75.9%
	post-graduate degree and above	8.3%	10.4%	11.7%	9.5%
disposable income	2000 yuan and below	7.9%	8.3%	19.0%	9.5%
	2001–4000 yuan	25.0%	20.0%	20.1%	24.6%
	4001–6000 yuan	34.7%	23.0%	21.2%	34.9%
	6001 yuan and above	32.4%	48.7%	39.6%	31.0%
BMI	≤18.4	17.6%	12.6%	14.7%	14.2%
	18.5–23.9	69.9%	70.9%	67.7%	73.7%
	≥24.0	12.5%	16.5%	17.6%	12.1%
Valid sample size		216	230	273	232

## Data Availability

All data included in this study are available upon request by contact with the corresponding author.
